# Is body size at birth related to circadian salivary cortisol levels in adulthood? Results from a longitudinal cohort study

**DOI:** 10.1186/1471-2458-10-346

**Published:** 2010-06-17

**Authors:** Per E Gustafsson, Urban Janlert, Töres Theorell, Anne Hammarström

**Affiliations:** 1 Dept of Public Health and Clinical Medicine, Family Medicine, Umeå University, Umeå, Sweden; 2 Dept of Public Health and Clinical Medicine, Epidemiology and Global Health, Umeå University, Umeå, Sweden; 3 Stress Research Institute, Stockholm University, Stockholm, Sweden

## Abstract

**Background:**

The hypothesis of fetal origins of adult disease has during the last decades received interest as an explanation of chronic, e.g. cardiovascular, disease in adulthood stemming from fetal environmental conditions. Early programming and enduring dysregulations of the hypothalamic-pituitary-adrenal (HPA axis), with cortisol as its end product, has been proposed as a possible mechanism by which birth weight influence later health status. However, the fetal origin of the adult cortisol regulation has been insufficiently studied. The present study aims to examine if body size at birth is related to circadian cortisol levels at 43 years.

**Methods:**

Participants were drawn from a prospective cohort study (n = 752, 74.5%). Salivary cortisol samples were collected at four times during one day at 43 years, and information on birth size was collected retrospectively from delivery records. Information on body mass during adolescence and adulthood and on health behavior, medication and medical conditions at 43 years was collected prospectively by questionnaire and examined as potential confounders. Participants born preterm or < 2500 g were excluded from the main analyses.

**Results:**

Across the normal spectrum, size at birth (birth weight and ponderal index) was positively related to total (area under the curve, AUC) and bedtime cortisol levels in the total sample. Results were more consistent in men than in women. Descriptively, participants born preterm or < 2500 g also seemed to display elevated evening and total cortisol levels. No associations were found for birth length or for the cortisol awakening response (CAR).

**Conclusions:**

These results are contradictory to previously reported negative associations between birth weight and adult cortisol levels, and thus tentatively question the assumption that only low birth weight predicts future physiological dysregulations.

## Background

During the last decades the hypothesis of fetal origins of adult disease has been proposed as a complement to social, behavioral or genetic etiological models [1]. The hypothesis postulates that propensity to chronic diseases in adulthood can be partially explained by a hazardous pre-and peri-natal environment, such as fetal undernutrition or inappropriate demands ex utero due to preterm birth [1,2]. Such harsh metabolic circumstances are thought to result in enduring adaptational changes in key physiological systems, a process called programming, conferring increased risks for diseases in adulthood [3]. The hypothesis has been corroborated in a number of studies, where markers of inadequate fetal conditions (e.g., low birth weight) have been shown to predict a number of common diseases and risk factors, such as hypertension, obesity and insulin resistance [4-6]. Both preterm birth and small body size at birth have been shown to be important predictors for later development of disease [2].

The hypothesis of fetal origins of adult disease presents theoretical challenges to models of cardiovascular and metabolic disease etiology and thereby might carry considerable implications for public health strategies directed at prevention of these diseases. Nevertheless, the mechanisms by which the intrauterine milieu influences the development of disease decades later remain to be elucidated. Programming of physiological stress systems, in particular the hypothalamus-pituitary-adrenal (HPA) axis, has been put forward as playing a central pathophysiological role [7,8]. The HPA axis is a neuroendocrine circuit constituting a part of the physiological stress system, with its main end product cortisol exerting wide-ranging metabolic, cardiovascular, immunological and central nervous effects [9]. Basal HPA axis activity and corresponding cortisol levels follow a circadian rhythm, with a sharp increase upon awakening (the cortisol awakening response, CAR) [10] and decreasing levels during the day, reaching low levels in the afternoon and evening. The HPA axis has the capacity for long-term dysregulations in face of early insults [8], with the development of metabolic and cardiovascular disease as a potential consequence in the long run [11,12].

Considering that enduring HPA axis dysregulations are hypothesized to represent early features of the causal chain, clarifying the role the HPA axis in the fetal origins of adult disease at an age when the cardiovascular morbidity and mortality rates are still low could be particularly valuable. However, only a handful of studies during the last decade have examined the association between body size at birth (mainly birth weight) and circadian cortisol levels in adulthood, and a number of methodological issues preclude any definitive conclusions about the fetal origins of the adult cortisol regulation. In general, studies have indicated that body size at birth might be inversely related to the cortisol secretion in adulthood [13-17], while a number of studies have reported no association [18-21], or that the association might be dependent on gestational age [22]. As pointed out in a meta-analysis by van Montfoort et al. [23], the majority of studies on the subject are based on small samples and might thus be underpowered; indeed, a pooled data analysis confirmed an inverse relationship between birth weight and (morning) cortisol levels [23]. Several studies have comprised samples of either women [19] or men [16]. Moreover, only a few small-sized studies have allowed for examination of time-of-day effects [15,19], while the larger studies in adults have focused solely on morning cortisol levels [14,16,17,22] or total cortisol secretion as assessed by urinary free cortisol [24]. Since the regulation of the HPA axis is believed to differ with respect to the circadian rhythm [10], and dysregulations might be restricted to specific parts of the circadian rhythm [25,26], this might possibly explain some of the inconsistent findings in previous research.

In conclusion, the fetal influences on the cortisol secretion in adulthood have so far been insufficiently studied, which is regrettable since the issue might give clues to the pathophysiology of cardiovascular disease in adulthood. To address these shortcomings, the present study, a prospective cohort study with retrospective record-based data collection of body size at birth, was undertaken. The aim was to examine if body size at birth is related to the circadian cortisol secretion at 43 years.

## Methods

### Sample

The sample was drawn from a 27-year prospective cohort study, the Luleå cohort, and comprises all pupils in ninth grade of the Swedish compulsory school living in Luleå in 1981, when the participants where 16 years of age (N = 1083; 506 girls and 577 boys), with follow-up surveys conducted in 1983, 1986, 1995 and 2008. The cohort has in various comparisons been found to be representative of the Swedish population [27]. Of the original cohort, there were 1071 subjects still alive in 2008, of which 1003 (93.7%) agreed to participate. Due to non-response on one or more key measures (see below), the effective sample size of the present report is N = 752 (74.5%). The study was approved by the Regional Ethical Review Board at Umeå University and informed consent was provided by all participants. For more details about the study, see Hammarström & Janlert [28].

### Procedures

The participants were investigated in 1981, 1983, 1986, 1995 and 2008 with a comprehensive questionnaire, focusing on the main areas of social, economic and work-related circumstances, health and health behavior and leisure activities.

In 2008, delivery records of the participants' mothers from 1965 (i.e., pertaining the participants' own birth) were retrieved from the archives of the respective delivery ward. From the delivery records, information on birth weight, birth length, date of delivery, date for last menstruation prior to delivery, and diagnoses according to the Swedish adaptation of the 6th revision of the International Classification of Disease [29], was documented.

In 2008, the participants completed one-day sampling of saliva. Salivary cortisol entails non-invasive sample collection and is highly correlated with the free and biologically active fraction of serum cortisol [30]. Sampling instructions and plastic Salivette tubes were distributed to all participants by mail. Saliva samples were collected by the participants themselves during one week-day at four time points: at awakening (sample 1), 15 minutes post-awakening (sample 2), before lunch (sample 3) and at bedtime (sample 4). No eating, drinking, tobacco use or physical exercise was allowed the hour before sampling. Date and time for each sample were recorded by the participants. Samples were mailed to the laboratory at the Umeå University Hospital at the latest the day after they were collected. Upon arrival to the laboratory, samples were centrifuged and stored until analysis. A commercial radioimmunoassay kit (Spectria Cortisol RIA, Orion Diagnostica) was used to estimate cortisol concentrations (nmol/L), accordingly to the routines of the laboratory. Analyses were performed on single samples. Total precision was C.V. = 12% at 89 nmol/L and C.V. = 7.1% at 11 nmol/L, and the detection limit 0.7 nmol/L.

In 1981 and 2008 the participants were weighed and their height was measured by trained medical personnel. In 1986 and 1995, self-reported height and weight were collected by the questionnaire.

### Measures

#### Exposure: body size at birth

Birth weight (BW) (g), Birth length (BL) (cm), and ponderal index (PI) (weight/length^3^, kg/m3) were used as the main continuous measures of body size at birth. There were 990 valid cases for BW (92.4%) and 983 cases with available BL and ponderal index (91.8%).

Gestational age was calculated as the time (weeks) between the last menstruation before delivery and the delivery, and all cases with a diagnosis of immaturity of the neonate were regarded as born preterm. Gestational age (GA) or diagnosis was only recorded for 657 cases in the delivery records. To preserve the sample size, a two-step procedure was therefore applied in the operationalization of preterm birth. First, all cases with a GA < 37 weeks and/or a diagnosis of immaturity was defined as preterm (n = 47/657; 7.2%). Second, a similar proportion of cases (7.2%) not classified as preterm in the first step, were classified as preterm based on birth weight, resulting in those with a birth weight < 2500 g being proxied as preterm birth. Of those with birth weight < 2500 g and with available information on gestational age and/or diagnosis, 89.7% were born before week 37 or had been diagnosed with immaturity. The correlation between the measured (preterm/diagnosis) and proxied (birth weight < 2500 g) variables were r = .70 (p < .001). Thus, all cases with a birth weight < 2500 g, as well as those of preterm birth or diagnosed immaturity were classified as preterm, n = 75 in the total sample and n = 60 in the effective sample (see Table 1). This variable is referred to in the manuscript as 'preterm or < 2500 g' or 'preterm or low birth weight'.

**Table 1:** Descriptive statistics of birth size and cortisol measures by sex, and difference between women and men (t test and χ^2 ^test)

Measure	Women		Men		Total		
	M (s)	N	M (s)	N	M (s)	N	P value*
Birth size							
Birth weight(g)	3281 (530)	372	3469 (598)	380	3376 (573)	752	< .001
Birth length(cm)	50.5 (2.2)	366	51.0 (2.5)	379	50.7 (2.4)	745	.002
Ponderal Index (kg/m^3^)	25.5 (2.4)	366	26.0 (2.9)	379	25.8 (2.7)	745	.008
Preterm or < 2500 g, n (%)	27 (7.3%)	372	33 (8.7%)	380	60 (8.0%)	752	.471
Cortisol at 43y							
AUC (nmol/L)	3.89 (1.6)	367	4.51 (1.6)	378	4.19 (1.6)	745	< .001
CAR (relative increase)	1.37 (1.6)	358	1.38 (1.7)	364	1.38 (1.7)	722	.861
Bedtime (nmol/L)	1.56 (2.0)	372	1.79 (2.1)	380	1.67 (2.0)	752	.007

For birth size measures, arithmetic means (s) are presented, and for cortisol measures, geometric means (s)

AUC = Area Under the Curve of diurnal cortisol, CAR = Cortisol Awakening Response

#### Outcome: salivary cortisol at 43 years

There were 761 cases with at least one valid cortisol sample and 755 cases with complete cortisol data. Due to skewed distributions of the cortisol concentrations (nmol/L) they were transformed by the natural logarithm before analysis to achieve normal distribution. From these log transformed cortisol concentrations, three cortisol variables were constructed as our main dependent measures, to represent total HPA axis activity as well as activity at different parts of the circadian rhythm (see Gustafsson et al. [31] for more details). 1) The area under the curve (AUC) was calculated with respect to ground [32] from sample 2-4, as an approximation of the total circadian cortisol output (log nmol/L × hours). The AUC was weighted by time by dividing with the total time between sample 2 and 4, yielding the unit log nmol/L. The AUC with respect to increase [32] was also calculated for complementary analyses. 2) The cortisol awakening response (CAR) was calculated as the log transformed concentrations at time 2-time 1 (dimensionless, equaling the log transformed ratio of sample 2 and sample 1), representing the cortisol response to awakening [10]. 3) Bedtime cortisol levels correspond to log transformed concentrations of sample 4 and (log nmol/L) representing cortisol levels at a period of normally low HPA axis activity. Since there was a large variation in sampling times, and cortisol levels change rapidly in the early morning, participants with a time interval < 10 minutes or > 60 minutes between time 1 and 2 were excluded from the calculation of CAR.

#### Potential confounders

Self-reported health behavior at the 2008 survey was examined as potential confounder: smoking (yes/no), snuff use (yes/no), alcohol consumption (> 80^th ^percentile). Alcohol consumption (gram pure alcohol consumption per year) was estimated from questions on the frequency of alcoholic beverage consumption, and approximate volume of beverage per occasion (separate items for beer, wine and spirits). Since body mass over the life course might be related to both birth weight [33] and to cortisol [34], body mass index (BMI) was also regarded as a potential confounder. BMI (kg/m^2^) was calculated for the participants at age 16, 43 (measured weight and height), 21 and 30 (self-reported height and weight). Since BMI at 30y and 43y were highly correlated (r = .79), BMI at 30y was excluded from the analyses to reduce multicollinearity effects.

In addition, a number of medical conditions or medications that could possibly affect cortisol secretion were identified. Diabetes, depression and asthma was based on a question on the presence of different medical conditions during the last 12 months, with the response options 'No', 'Yes, modest' and 'Yes, severe'. A positive response was considered presence of diabetes (n = 13). Only 'severe' was considered presence of depression (n = 39) and asthma (n = 7). Participants were also asked about the use of oral glucocorticoids (n = 6), contraceptives (n = 84) and medications against depression (n = 45) and diabetes (n = 7). In total, 125 women and 35 men had either condition or used any medication. Menstrual cycle phase (n = 233) was calculated from self-reported days since last menstruation start at the time of saliva sampling; 0-14 days (follicular phase, n = 128) and 15-32 days (luteal phase, n = 105).

### Data analysis

Missing data was mainly due to drop-out on the cortisol measures. Those without cortisol data (n = 238) did not differ in body size at birth from those with available cortisol data (n = 752) (t test; p > .11). There were n = 752 cases (372 women, 380 men) available with at least one valid cortisol measure and birth weight information, comprising the effective sample. For descriptive statistics, see Table 1.

Since the relationship between birth size and cortisol levels in adulthood might be confounded by prematurity [22], preterm or low birth weight (< 2500 g) participants as defined by the proxy variable (n = 60) were excluded from the main analyses and examined separately in explorative analyses. These analyses were rerun on the subsample with known preterm status (i.e., recorded presence or absence of preterm birth or diagnosed immaturity), excluding those of preterm birth or with diagnosis of immaturity (n = 458). These analyses lead to similar results as the analyses on the inclusive sample, excluding those with proxied preterm status (data not shown). Analyses were also rerun excluding participants with self-reported presence of any medical condition (asthma, diabetes, depression) or using any medication (oral glucocorticoids, contraceptives, or medication against diabetes or depression), yielding similar results to analyses on the total sample (data not shown). To maintain the sample size, only the results from the inclusive sample are therefore reported in the results section. Complementary analyses included the potential confounders BMI during the life course and current health behavior. Due to missing data on one or more of the potential confounders, in the multivariable analyses the n dropped further, lowest in analyses simultaneously including body size, CAR and all confounders (n = 629). Information on menstrual cycle phase was only available for 233 women; exploratory analyses (t tests) revealed no significant differences in cortisol measures or birth weight (all ps > .20) between women in follicular and luteal phase (data not shown).

Linear regression analysis was used in the main analyses to assess the influence of size at birth on cortisol measures at 43 years, separately for each cortisol measure, with the subsequent addition of potential confounders. The alternative AUC measure (with respect to increase) was significantly predicted by BW (p = .006) and PI (p = .030) similarly to AUC with respect to ground; only results concerning AUC with respect to ground are therefore reported in the results section.

To aid the interpretation of the regression coefficients, kilogram was used instead of gram as the unit of measurement for birth weight in the regression models. Regression estimates are reported as anti-logged unstandardized coefficients (b) and standardized coefficients (β). For the purpose of graphical presentation, cases were categorized into groups based on birth weight intervals of 500 g; < 2500 g or preterm birth (n = 60), 2500-2999 g (n = 118), 3000-3499 g (n = 248), 3500-3999 g (n = 229), and ≥4000 (n = 97).

Analyzes were conducted on the whole sample as well as on women and men separately. SPSS v17.0 was used for all analyses.

## Results

### Relationships among body size and cortisol measures

Men had larger body size at birth than women and higher total (AUC) and bedtime cortisol levels at age 43 (Table 1). The body size measures were, as expected, correlated; BW correlated strongly with BL (r = .79) and PI (r = .55), while BL was not related to PI (r = -.06). Correspondingly, AUC correlated strongly with bedtime cortisol (r = .70) and weakly with CAR (r = .11), while there was no association between bedtime cortisol and CAR (r = -.03).

### Normal ranges of body size at birth and cortisol levels

Regressing AUC on body size at birth measures indicated a positive relationship between body size measures and total cortisol secretion (see Table 2). In the total sample, AUC was significantly predicted by BW (R^2 ^= 1.7%) and PI (R^2 ^= 0.9%), but not by BL. One kilogram increase in birth weight corresponded to a 13% increase in AUC. Similar patterns for birth weight was observed in men, while in women this association was of weaker magnitude and not significant.

**Table 2:** Summary of separate regression models: cortisol area under the curve (AUC, log nmol/L) on body size at birth (birth weight, birth length, ponderal index); b = anti-logged unstandardized regression coefficient, β = standardized regression coefficients

Sample	Criterion: Cortisol AUC				
Predictor					
	**Model estimates**		**Predictor estimates**		
	**N**	**R^2^**	**b**	**β**	**p**

Total sample					
Birth weight (kg)	685	.017	1.13	.13	< .001
Birth length (cm)	683	.005	1.02	.07	.065
Ponderal index (kg/m^3^)	683	.009	1.02	.10	.012
Women					
Birth weight (kg)	340	.006	1.08	.08	.147
Birth length (cm)	338	.000	1.00	.02	.763
Ponderal index (kg/m^3^)	338	.008	1.02	.09	.106
Men					
Birth weight (kg)	345	.015	1.12	.12	.022
Birth length (cm)	345	.006	1.02	.08	.143
Ponderal index (kg/m^3^)	345	.005	1.01	.07	.181

Similar analyses were performed with CAR and bedtime cortisol as criterion variables, to examine specific influences on distinct parts of the circadian cortisol rhythm. While there were no significant relationships between body size at birth and CAR (data not shown, all ps > .10), positive relationships were found for bedtime cortisol levels (see Table 3), similarly to the models including AUC. In the total sample, both BW (R^2 ^= 1.3%) and PI (R^2^= 1.5%) appeared to exert influences on bedtime cortisol levels; one kilogram increase in birth weight involved 19% increase in bedtime cortisol levels. As with the AUC models, similar results for birth weight was observed in men, but were weaker and not significant in women.

**Table 3:** Summary of separate regression models with bedtime cortisol levels (log nmol/L), on body size at birth (birth weight, birth length, ponderal index); b = anti-logged unstandardized regression coefficient, β = standardized regression coefficients

Sample	Criterion: bedtime cortisol levels				
Predictor					
	**Model estimates**		**Predictor estimates**		
	**N**	**R^2^**	**b**	**β**	**p**

Total sample					
Birth weight (kg)	692	.013	1.19	.11	.003
Birth length (cm)	690	.001	1.01	.03	.390
Ponderal index (kg/m^3^)	690	.015	1.03	.12	.001
Women					
Birth weight (kg)	345	.004	1.11	.06	.240
Birth length (cm)	343	.000	0.99	-.02	.682
Ponderal index (kg/m^3^)	343	.012	1.03	.11	.041
Men					
Birth weight (kg)	347	.015	1.21	.12	.021
Birth length (cm)	347	.003	1.02	.05	.349
Ponderal index (kg/m^3^)	347	.012	1.03	.11	.041

Adding BMI at age 16, 21 and 43 and health behavior at age 43 did not influence the findings substantially (data not shown); in the total sample the regression coefficients were still significant for both BW (p < .001) and PI (p = .019) in the AUC models, and for both BW (p < .001) and PI (p < .001) in the bedtime cortisol models. Similarly, regression coefficients were still significant after excluding cases with medical conditions, medication or contraceptives (data not shown); for BW (p < .001) and PI (p = .025) in the AUC models, and for BW (p < .001) and PI (p < .001) in the bedtime cortisol models.

### Preterm birth and cortisol levels

**Figure 1:**
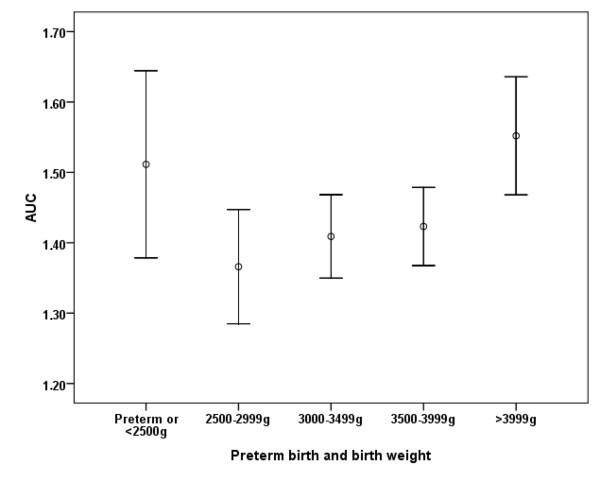
**Area under the curve (AUC, log nmol/L, mean and 95% CI) by intervals of birth weight, and preterm or < 2500 g birth**.

**Figure 2:**
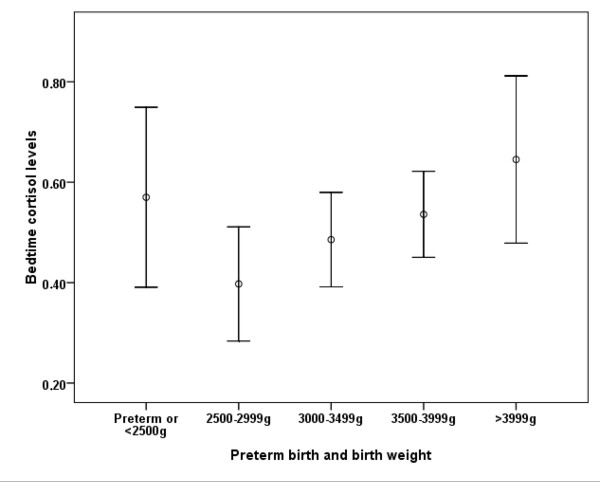
**Bedtime cortisol levels (log nmol/L, mean and 95% CI) by intervals of birth weight, and preterm or < 2500 g birth**.

A descriptive exploration of the excluded preterm or low birth weight (< 2500 g) participants (n = 60) indicated that this group displayed high AUC and bedtime cortisol levels at similar levels as the groups with large body size at birth. As can be seen in Figure 1 (AUC) and Figure 2 (Bedtime cortisol levels), this resulted in a curvilinear relationship between BW and cortisol measures when including the entire sample.

## Discussion

The main finding of this study is that birth weight and ponderal index are related to total cortisol levels at 43 years, which seemed to be explained by increased levels during the latter part of the day, independently of current health behavior and body mass over the life course and unaffected by the exclusion of participants with medical conditions and medications. Interestingly though, while most studies have found birth weight to be negatively related to cortisol levels in adulthood [15-17,23], we found the opposite direction of association: higher cortisol levels by larger body size at birth, across the normal spectrum of birth weight. This unexpected finding calls for some elaboration as to why our results would differ so substantially from previous findings.

First, most studies on the subject have been performed on small samples and a considerably higher drop-out rate which could introduce selection bias and misestimation of associations, compared to our population-based sample with high participation rate. Second, most studies on adults, and all with larger sample size (e.g., [17]), have not specifically examined cortisol levels during the latter part of the day, which in our analyses was the period of the circadian rhythm most prominently related to birth weight. Rosmalen and co-workers [35] have reported no substantial influence of birth weight on either morning or evening cortisol levels, but their large sample consisted of 10-12-year-old children, so their findings might not be directly generalizable to adults. Third, important sample characteristics (e.g. age range at the time of cortisol measurement) differ considerably between studies, rendering direct comparisons of dubious validity. However, yet another issue might be helpful for understanding the discrepancies in results: the management of preterm or low birth weight cases. In an explorative examination, preterm birth or low birth weight (< 2500 g) seemed to be related to higher bedtime cortisol levels of similar magnitude as of the group with the highest birth weight. This suggests that increased basal HPA axis activity might be produced by either harsh pre- or perinatal metabolic circumstances, or in the normal spectrum, also by supposedly favorable circumstances. Discrepant associations for preterm children compared to mature birth children in relation adult cortisol levels have been reported previously by Kajantie et al. [22], who found a positive relationship in children born at a later gestational age (> 39 weeks), but a negative association in those born early at term (37-39 weeks). A U-shaped association between birth weight and 24-hour glucocorticoid metabolites has also been described in preadolescent children [36]. Although these studies differs from the present one on major methodological points, it corroborates the possibility of a positive relationship between birth size and adult cortisol levels in those maturely born, and a U-shaped association across the entire range of maturity and gestational age at birth.

This finding may warrant a re-evaluation of the hypothesis that only low birth weight might confer health-related physiological dysregulation in adulthood. Elevated evening cortisol levels have been found in men with coronary artery disease, correlated with markers of inflammation [37] and has also been linked to morbidity of chronic diseases as well as to mortality rates in women, but not in men [38], indicating that normal but high evening cortisol levels might confer increased health risks and thus emphasizing the potential public health importance of our findings. Although the associations reported in this study were rather weak, the long-term association with measure points 43 years apart would suggest that the observed dysregulations are enduring and stable, which could exert a health-damaging effect possibly over the entire life course. Moreover, even small effects might be of public health importance when generalized to the general population.

Although there is evidence of health-damaging consequences of high evening cortisol levels, the possibility of high cortisol levels representing a healthy pattern and low cortisol levels representing potentially harmful hypocortisolism [39] has to be considered. Hence, our findings cannot be unambiguously interpreted as representing an increased health risks with increasing birth weight, although we also found numerically high cortisol concentrations in the preterm or low birth < 2500 g subgroup- a group with well-established health risks across the life course [2,40]. Since the fetal origins hypothesis examining outcomes such as cardiovascular disease has found linear relationships between birth weight and health measures, the pattern of a U-shaped relationship to cortisol seem to be contradictory. Speculatively, it is possible that different mechanisms operate in the normal spectrum of maturity as compared to those of preterm birth or of low birth weight, with different physiological trajectories yielding apparently similar outcomes in adulthood. Nevertheless, the significance of increased cortisol levels for these groups remains to be elucidated.

The analyses on women and men separately entailed lower power compared to the analyses on the total sample, as indicated by the less reliable findings, and should therefore be interpreted with caution. However, the separate analyses suggest that the influence of birth weight on both total (AUC) and bedtime cortisol levels was largely driven by corresponding associations among men, while these associations were considerably weaker and non-significant among women. As pointed out in a recent review [41], low birth weight is more consistently related to an increased HPA axis reactivity to acute stressors in men than in women, while there seems to be a greater influence on autonomic nervous system response in women. Thus, our results indicate that the circadian HPA axis regulation, in addition to the HPA response to acute challenges, might be more sensitive for early life influences in men than in women.

We did not find any evidence for an effect on the cortisol levels during the earlier part of the circadian rhythm. As described in the introduction, research focusing on parts of the circadian rhythm has almost exclusively studied cortisol levels during the early morning, and perhaps this is a partial reason why the literature on fetal origins of adult cortisol levels is abundant with null findings. There could also be methodological explanations to our null findings concerning CAR (see below).

### Methodological considerations

Major strengths of the study include a large representative sample with low drop-out rate, and the saliva sampling protocol including assessment at different part of the day. Although there was a considerably larger drop-out on the cortisol measures, there was no evidence for selection bias with respect to birth weight.

Saliva was sampled only during one day. This would be expected to contribute to measurement error and attenuation of observed relationships. Sampling over several days would have been a preferred method. Since cortisol levels peak approximately 30 min post-awakening, our lack of findings regarding the cortisol awakening response could possibly be attenuated by the sampling protocol of only 15 min between morning samples. Moreover, since we have no information regarding the accuracy of reported sampling time, non-adherence could introduce random error and attenuation of estimated effects [42]. Although the cortisol levels would not be expected to be influenced greatly by minor variations in sampling time during the later part of the day, the estimation of CAR is sensitive to such non-adherence since cortisol levels change very rapidly during this part of the circadian rhythm. For example, even a short time interval between awakening and the first sample could greatly influence the estimation of CAR [43,44]. Although time of awakening was not reported by the participants, self-reported sampling time might not be an optimal method [43]. These issues could possibly be methodological explanations of our negative findings regarding cortisol levels in the morning. While objective measures of the time of awakening and sampling would have been an optimal method to reliably assess the CAR, such high-constraint methods might be difficult and costly to implement in large population-based samples.

## Conclusions

In those not born preterm or with low birth weight, birth size and ponderal index are positively related to circadian cortisol levels during the later part of the day in mid-adulthood. Similarly high cortisol levels also seem to be present in those of preterm birth or of low birth weight. Together, these findings indicate that elevated cortisol levels might be a consequence of both ends of the maturity birth spectrum; an issue that has to be further examined in future research. Moreover, the pathophysiological significance of such relative hypercortisolism has to be elucidated, with the aim of understanding the mechanisms by which fetal circumstances influence health later in life.

## Competing interests

The authors declare that they have no competing interests.

## Authors' contributions

AH was responsible for the conception and design of the study and UJ and TT contributed to the design of the study. AH and UJ were responsible for the data collection. PEG was responsible for the statistical analyses and writing the first draft of the manuscript. All authors participated in interpreting the results, provided input on manuscript drafts, and read and approved the final manuscript.

## Pre-publication history

The pre-publication history for this paper can be accessed here:

http://www.biomedcentral.com/1471-2458/10/346/prepub

## References

[B1] Barker DJ The fetal and infant origins of adult disease BMJ 1990 301 1111 10.1136/bmj.301.6761.1111 2252919 PMC1664286

[B2] Hack M Adult outcomes of preterm children J Dev Behav Pediatr 2009 30 460 470 10.1097/DBP.0b013e3181ba0fba 19823140

[B3] Barker DJ Fetal origins of coronary heart disease BMJ 1995 311 171 174 7613432 10.1136/bmj.311.6998.171 PMC2550226

[B4] Curhan GC Willett WC Rimm EB Spiegelman D Ascherio AL Stampfer MJ Birth weight and adult hypertension, diabetes mellitus, and obesity in US men Circulation 1996 94 3246 3250 8989136 10.1161/01.cir.94.12.3246

[B5] Curhan GC Chertow GM Willett WC Spiegelman D Colditz GA Manson JE Speizer FE Stampfer MJ Birth weight and adult hypertension and obesity in women Circulation 1996 94 1310 1315 8822985 10.1161/01.cir.94.6.1310

[B6] Grunnet L Vielwerth S Vaag A Poulsen P Birth weight is nongenetically associated with glucose intolerance in elderly twins, independent of adult obesity J Intern Med 2007 262 96 103 10.1111/j.1365-2796.2007.01793.x 17598817

[B7] Edwards CR Benediktsson R Lindsay RS Seckl JR Dysfunction of placental glucocorticoid barrier: link between fetal environment and adult hypertension? Lancet 1993 341 355 357 10.1016/0140-6736(93)90148-A 8094124

[B8] Phillips DI Programming of the stress response: a fundamental mechanism underlying the long-term effects of the fetal environment? J Intern Med 2007 261 453 460 10.1111/j.1365-2796.2007.01801.x 17444884

[B9] Sapolsky RM Romero LM Munck AU How do glucocorticoids influence stress responses? Integrating permissive, suppressive, stimulatory, and preparative actions Endocr Rev 2000 21 55 89 10.1210/er.21.1.55 10696570

[B10] Wilhelm I Born J Kudielka BM Schlotz W Wust S Is the cortisol awakening rise a response to awakening? Psychoneuroendocrinology 2007 32 358 366 10.1016/j.psyneuen.2007.01.008 17408865

[B11] Rosmond R Bjorntorp P The hypothalamic-pituitary-adrenal axis activity as a predictor of cardiovascular disease, type 2 diabetes and stroke J Intern Med 2000 247 188 197 10.1046/j.1365-2796.2000.00603.x 10692081

[B12] McEwen BS Protective and damaging effects of stress mediators N Engl J Med 1998 338 171 179 10.1056/NEJM199801153380307 9428819

[B13] Szathmari M Vasarhelyi B Tulassay T Effect of low birth weight on adrenal steroids and carbohydrate metabolism in early adulthood Horm Res 2001 55 172 178 10.1159/000049991 11598370

[B14] Phillips DI Walker BR Reynolds RM Flanagan DE Wood PJ Osmond C Barker DJ Whorwood CB Low birth weight predicts elevated plasma cortisol concentrations in adults from 3 populations Hypertension 2000 35 1301 1306 10856281 10.1161/01.hyp.35.6.1301

[B15] Fall CH Dennison E Cooper C Pringle J Kellingray SD Hindmarsh P Does birth weight predict adult serum cortisol concentrations? Twenty-four-hour profiles in the United kingdom 1920-1930 Hertfordshire Birth Cohort J Clin Endocrinol Metab 2002 87 2001 2007 10.1210/jc.87.5.2001 11994332

[B16] Phillips DI Barker DJ Fall CH Seckl JR Whorwood CB Wood PJ Walker BR Elevated plasma cortisol concentrations: a link between low birth weight and the insulin resistance syndrome? J Clin Endocrinol Metab 1998 83 757 760 10.1210/jc.83.3.757 9506721

[B17] Power C Li L Hertzman C Associations of early growth and adult adiposity with patterns of salivary cortisol in adulthood J Clin Endocrinol Metab 2006 91 4264 4270 10.1210/jc.2006-0625 16912134

[B18] Dalziel SR Parag V Rodgers A Harding JE Cardiovascular risk factors at age 30 following pre-term birth Int J Epidemiol 2007 36 907 915 10.1093/ije/dym067 17468503

[B19] Kajantie E Eriksson J Osmond C Wood PJ Forsen T Barker DJ Phillips DI Size at birth, the metabolic syndrome and 24-h salivary cortisol profile Clin Endocrinol (Oxf) 2004 60 201 207 10.1046/j.1365-2265.2003.01965.x 14725681

[B20] Reynolds RM Walker BR Syddall HE Andrew R Wood PJ Whorwood CB Phillips DI Altered control of cortisol secretion in adult men with low birth weight and cardiovascular risk factors J Clin Endocrinol Metab 2001 86 245 250 10.1210/jc.86.1.245 11232008

[B21] Ward AM Syddall HE Wood PJ Chrousos GP Phillips DI Fetal programming of the hypothalamic-pituitary-adrenal (HPA) axis: low birth weight and central HPA regulation J Clin Endocrinol Metab 2004 89 1227 1233 10.1210/jc.2003-030978 15001615

[B22] Kajantie E Phillips DI Andersson S Barker DJ Dunkel L Forsen T Osmond C Tuominen J Wood PJ Eriksson J Size at birth, gestational age and cortisol secretion in adult life: foetal programming of both hyper- and hypocortisolism? Clin Endocrinol (Oxf) 2002 57 635 641 10.1046/j.1365-2265.2002.01659.x 12390338

[B23] van Montfoort N Finken MJ le Cessie S Dekker FW Wit JM Could cortisol explain the association between birth weight and cardiovascular disease in later life? A meta-analysis Eur J Endocrinol 2005 153 811 817 10.1530/eje.1.02050 16322386

[B24] Kajantie E Feldt K Raikkonen K Phillips DI Osmond C Heinonen K Pesonen AK Andersson S Barker DJ Eriksson JG Body size at birth predicts hypothalamic-pituitary-adrenal axis response to psychosocial stress at age 60 to 70 years J Clin Endocrinol Metab 2007 92 4094 4100 10.1210/jc.2007-1539 17848405

[B25] Ranjit N Young EA Kaplan GA Material hardship alters the diurnal rhythm of salivary cortisol Int J Epidemiol 2005 34 1138 1143 10.1093/ije/dyi120 15951357

[B26] Pico-Alfonso MA Garcia-Linares MI Celda-Navarro N Herbert J Martinez M Changes in cortisol and dehydroepiandrosterone in women victims of physical and psychological intimate partner violence Biol Psychiatry 2004 56 233 240 10.1016/j.biopsych.2004.06.001 15312810

[B27] Hammarström A Youth unemployment and ill-health. results from a two year follow-up study. (in Swedish, summary in English) Doctoral thesis, monograph 1986 Karolinska Institute

[B28] Hammarstrom A Janlert U Health selection in a 14-year follow-up study--a question of gendered discrimination? Soc Sci Med 2005 61 2221 2232 10.1016/j.socscimed.2005.04.020 16099575

[B29] Sverige. Medicinalstyrelsen Klassifikation av sjukdomar: International statistical classification of diseases, injuries and causes of death: 1955 revision adapted for indexing of hospital records and morbidity statistics 1964 6 Stockholm: Nord Bokh

[B30] Kirschbaum C Hellhammer DH Salivary cortisol in psychoneuroendocrine research: recent developments and applications Psychoneuroendocrinology 1994 19 313 333 10.1016/0306-4530(94)90013-2 8047637

[B31] Gustafsson PE Janlert U Theorell T Hammarstrom A Life-course socioeconomic trajectories and diurnal cortisol regulation in adulthood Psychoneuroendocrinology 2010 35 4 613 623 10.1016/j.psyneuen.2009.09.019 19879057

[B32] Pruessner JC Kirschbaum C Meinlschmid G Hellhammer DH Two formulas for computation of the area under the curve represent measures of total hormone concentration versus time-dependent change Psychoneuroendocrinology 2003 28 916 931 10.1016/S0306-4530(02)00108-7 12892658

[B33] Kuh D Hardy R Chaturvedi N Wadsworth ME Birth weight, childhood growth and abdominal obesity in adult life Int J Obes Relat Metab Disord 2002 26 40 47 10.1038/sj.ijo.0801861 11791145

[B34] Pasquali R Vicennati V Cacciari M Pagotto U The hypothalamic-pituitary-adrenal axis activity in obesity and the metabolic syndrome ANYAS 2006 1083 111 128 10.1196/annals.1367.009 17148736

[B35] Rosmalen JG Oldehinkel AJ Ormel J de Winter AF Buitelaar JK Verhulst FC Determinants of salivary cortisol levels in 10-12 year old children; a population-based study of individual differences Psychoneuroendocrinology 2005 30 483 495 10.1016/j.psyneuen.2004.12.007 15721059

[B36] Clark PM Hindmarsh PC Shiell AW Law CM Honour JW Barker DJ Size at birth and adrenocortical function in childhood Clin Endocrinol (Oxf) 1996 45 721 726 10.1046/j.1365-2265.1996.8560864.x 9039338

[B37] Nijm J Kristenson M Olsson AG Jonasson L Impaired cortisol response to acute stressors in patients with coronary disease. Implications for inflammatory activity J Intern Med 2007 262 375 384 10.1111/j.1365-2796.2007.01817.x 17697159

[B38] Schoorlemmer RM Peeters GM van Schoor NM Relationships between cortisol level, mortality and chronic diseases in older persons Clin Endocrinol (Oxf) 2009 71 779 86 10.1111/j.1365-2265.2009.03552.x 19226268

[B39] Heim C Ehlert U Hellhammer DH The potential role of hypocortisolism in the pathophysiology of stress-related bodily disorders Psychoneuroendocrinology 2000 25 1 35 10.1016/S0306-4530(99)00035-9 10633533

[B40] Saigal S Doyle LW An overview of mortality and sequelae of preterm birth from infancy to adulthood Lancet 2008 371 261 269 10.1016/S0140-6736(08)60136-1 18207020

[B41] Kajantie E Raikkonen K Early life predictors of the physiological stress response later in life Neurosci Biobehav Rev in press 10.1016/j.neubiorev.2009.11.013 19931557

[B42] Kudielka BM Broderick JE Kirschbaum C Compliance with saliva sampling protocols: electronic monitoring reveals invalid cortisol daytime profiles in noncompliant subjects Psychosomatic medicine 2003 65 313 319 10.1097/01.PSY.0000058374.50240.BF 12652000

[B43] Dockray S Bhattacharyya MR Molloy GJ Steptoe A The cortisol awakening response in relation to objective and subjective measures of waking in the morning Psychoneuroendocrinology 2008 33 77 82 10.1016/j.psyneuen.2007.10.001 17996380

[B44] Okun ML Krafty RT Buysse DJ Monk TH Reynolds CF Begley A Hall M What constitutes too long of a delay? Determining the cortisol awakening response (CAR) using self-report and PSG-assessed wake time Psychoneuroendocrinology 2010 35 460 468 10.1016/j.psyneuen.2009.08.017 19762158 PMC2823961

